# Biological Risk in Italian Prisons: From the COVID-19 Management to the Development of a Standardized Model for Emergency Response

**DOI:** 10.3390/ijerph181910353

**Published:** 2021-10-01

**Authors:** Cristiano Franchi, Ezio Giacalone, Daniele Di Giovanni, Stefania Moramarco, Mariachiara Carestia

**Affiliations:** 1 Central Laboratory for the National DNA Database, Prison Administration Department, Ministry of Justice, 00146 Rome, Italy; 2 Prison and Probation Police, Ministry of Justice, 00100 Rome, Italy; ezio74@yahoo.com; 3 Department of Industrial Engineering, University of Rome Tor Vergata, 00133 Rome, Italy; daniele.di.giovanni@uniroma2.it; 4 School of Medicine and Surgery, Unicamillus-Saint Camillus International University of Health Sciences, 00131 Rome, Italy; 5 Department of Biomedicine and Prevention, University of Rome Tor Vergata, 00133 Rome, Italy; stefania.moramarco@uniroma2.it

**Keywords:** biological risk, communicable diseases, indoor facilities, prison system, epidemics, outbreaks, contingency plan, public health

## Abstract

Within the confinements of critical infrastructures, the COVID-19 pandemic is posing a series of challenges to Health Management. In the spotlight of highly contagious and quick spreading diseases within such enclosed facilities, whether it be a detention facility or otherwise, the health and safety of those living within its internment is paramount. This paper aims to highlight the specific challenges and the possible solutions to counteract this problem, starting from the lessons learnt from the Italian prison system case study. Following the general description of the available resources within the Italian prisons, the study aimed at specifically describing the first counteracting measures deployed by the Italian prison authorities during the first phase of the COVID-19 outbreak (February–July 2020). The aim was to propose an integrated plan capable of responding to a biological threat within the prisons. In particular, the study describes the actions and technical features that, in accordance with national and international legal frameworks and the relevant organisational bodies that run the Italian Prison Service, had been adopted in managing, right from the start, the COVID-19 pandemic until Summer 2020. Available information and data showed the ability of the prison administration to comply almost completely with WHO’s technical and human rights recommendations and also, in successfully handling prison emergencies both in terms of the sick and the deceased in line with the epidemiological framework of the general population. In addition, the paper proposes a draft of guidelines that should involve the National Health Service and the Prison Service that are aimed at supporting the local prison facilities with drawing up their own biological incident contingency plans. An approved, legal, standardised plan could increase the awareness of prison managers. It could even increase their self-confidence, in particular, with regard to cases of dispute and their ability to respond to them. In fact, it is valuable and forward-thinking to be able to demonstrate that every endeavour has been taken and that ‘certified’ best practices have been put in place in accordance with the national standards.

## 1. Introduction

Prisons and other places of detention (i.e., jails and migrants host centres) represent serious challenges in case of an outbreak: overcrowding, unsanitary conditions, poor nutrition and physical and psychological stress are specific issues that are likely to pose a greater risk of infection among inmates with respect to the outside society. Indeed, some authors evaluated that the infection rate in case of pandemic flu in prison could be as high as 90% [1]. Moreover, the proportion of inmates who suffer from diagnosed mental illness, drug abuse and alcoholism is significantly higher if compared to the general population and, according with WHO statistics, tuberculosis, human immunodeficiency virus (HIV) and Hepatitis B-C are over-represented in prisons and detention centres [2,3]. This evidence is probably due to the fact that the inmate population is not a representative sample of the general population. 

### 1.1. Infectious Diseases in Prison Environments

When managing the threat of communicable diseases in prison and in other places of detention, one must bear in mind that there are at least three aspects which differ from external society and its environment [4]:

(a) The infrastructure of the establishment, its age, its facilities (e.g., toilets, showers, canteen, catering, kitchens, open spaces and so on), the dimension of cells, the presence of green spaces, light, humidity, warming, ventilation etc.).
(b) The organizational features which refer, for example, to the accessibility to healthcare, the ratio between prison staff size and the number of detainees, the quality and quantity of food, the crowding of cells, the possibility of access to treatment activities, the frequency of visits and the preparedness of prison staff to manage biological emergency plans.
(c) The sub-population health condition is usually not overlapping the distribution curve referring to the general population: inmates are often affected mainly by prior diseases that involve blood borne viruses (e.g., HIV, hepatitis B and C), mainly [4]. Furthermore, the prison environment can represent the opportunity to deliver care, assistance, and medical screening to large sectors of a population that is often excluded.

### 1.2. The Threat of COVID-19 Pandemic in European Prisons

The SARS-CoV-2 virus has spread worldwide, including to prison facilities that have not been spared from emergency management. 

The pandemic spread and the effects in terms of the prison rate population have been described in the SPACE I report [5] for the Council of Europe. It refers to the member states’ prisons during the first nine months of the outbreak, from January 2020 to September 2020. The report indicates that:

(a) There has been a general decrease (more than 4%) in the incarceration rate in 20 state institutions among those 35 that participated in the entire initiative (three questionnaires fulfilled).
(b) 11 institutions registered no significant change (population rate was between +/−4%).
(c) In four institutions there was an increase (+4%) in prison population by mid-September 2020.

The variation in prison population rates is clearly due to:

(d) A direct effect of the deliberate release of inmates as a preventive measure to fight the SARS-CoV-2 spread in closed spaces.
(e) A possible side effect of general lockdowns that have been imposed on state populations with the consequence of a reduction of crimes, on one side, and of magistrates’ activities on the other (this is likely to be the case of what happened in Sweden, where no general lockdown was imposed).
(f) The report also collects the numbers of infected people among inmates and prison staff: in the 35 facilities that were able to deliver this information, there was a total amount of 3300 inmates and 5100 prison staff who had been infected by the middle of September 2020. Those figures seem to show that the rate of infection among inmates and prison staff was lower than in the general population during the first nine months of the pandemic.

The aim of this study is to frame the Italian prisons responsiveness to the SARS-CoV-2 in the wider European context and subsequently to:

(a) Report on the Italian preparedness and capability to manage the first wave of the COVID-19 pandemic in prison with reference to international standards and guidelines provided by WHO.
(b) Outline and propose a possible guideline for contingency plans in prison in case of a biological event. Such a proposal could open a debate on a “de iure condendo” preparedness tool for Italian prison administration.

## 2. Materials and Methods

### 2.1. Setting: Organization of the Italian Penitentiary System 

The Italian penitentiary system is under the responsibility of the Ministry of Justice —Department of Penitentiary Administration (DAP). Statistics reveal that the population of detainees and internes varies from a minimum of 52,164 to a maximum of 67,961 in the decade that spans from 2009 to 2019 [6]. Penitentiary administration is deployed between the central and the inter-regional offices, with mainly administrative duties, and the local facilities that are the prisons. Each establishment is managed by a governor who oversees the work of the four areas that constitute the penitentiary. The four areas are: (1) the security area, managed by the prison and probation police; (2) the re-education area, run by psychologists and social assistants; (3) the healthcare area, managed by the national healthcare system and (4) the secretariat that supports the governor for the bureaucratic matters.

After their arrival, prisoners are registered and searched by the prison and probation police. They are interviewed by a psychologist and visited by a general practitioner. Every inmate has the right to healthcare. In fact, the only prisoner right that is removed is the freedom of movement [7]. This right is guaranteed by the national healthcare system, locally managed by the A.S.L. (Azienda Sanitaria Locale—the local health authority) that depends upon the regional government. The ASL undertakes any examinations and medical tests concerning prevention, diagnosis, treatment, and rehabilitation. Furthermore, they provide a surveillance network for detection of communicable diseases for rapid medical action and epidemiological issues.

In the case of a suspected infectious disease, the medical practitioner who works in the prison is compelled to notify the ASL. The diseases requiring notification are listed in four main classes which have dedicated forms to fill [8]. 

### 2.2. Methods

At first, we focused on relevant documents, regarding the COVID-19 emergency, that were issued by the Italian government starting from 1 February 2020 until the following July (e.g., legislative decrees). We then consulted the very first circular letters provided by DAP to deploy specific counteracting measures to Italian prisons. All the searches were performed in government, police unions and human associations and other stakeholders’ websites. [9,10,11,12,13,14,15,16,17].

At a later stage, we compared the WHO’s “Preparedness, prevention, and control of COVID-19 in prison and other places of detention” (15 March 2020) advice and recommendations [18] with the previously released and aforementioned Italian provisions. 

The comparison between the “expected” (WHO guidance [18]) and the “observed” (government and administration provisions [9,10,11,12,13,14,15,16,17]) needed to broaden the legislative framework to the penitentiary law [19].

Complete and capillary data regarding infected, sick and dead inmates were not officially released by the prison service during the first months of the pandemic (February–August 2020), thereby we obtained some partial data only from the stakeholder’s papers. That is the case of Antigone’s XVI report on prison conditions [20] and of the SPACE 1 report [5]. Antigone is one of the most active and best-known no-profit associations that advocate for inmates’ rights and release publications periodically according to their monitoring activities on prison conditions in Italy. SPACE 1 (Statistiques Pénales Annuelles du Conseil de l’Europe) is a project issued by the University of Lausanne for the Council of Europe (47 members). The aim of the SPACE 1 project is to constantly monitor the use of prisons in the 47 state facilities.

The “top-down” approach proposed by WHO and utilised in some countries by some agencies was ultimately selected (e.g., PHE in England [21]) to elaborate and draft a new hypothetical Italian contingency plan guideline. 

## 3. Results

### 3.1. COVID-19 Pandemic Countermeasures

According to the Italian Prison Service guidelines [11,12,13,14,15,16], the main and most numerous directives that have been put in place, for COVID-19 in prison management, relate the so called “social distancing”, while a few actions have been engaged to inform prisoners and custodial staff about fundamentals of SARS-CoV-2 biology and medical issues and, to a lesser degree, to train them to properly use PPE and disinfectants (see Table 1). 

During the first phase of the outbreak, in the absence of any vaccines or effective medical countermeasures, the immediate response strategy relied on inter-personal distance, requiring that prisoners maintain a minimum distance of 1 m away from each other, and that they use PPE and disinfection to reduce the spread of virus particles. The main aspects of prison day life that have been modified regarded:

(a) Pre-triage and health isolation of new prisoners.
(b) The definition of COVID-19 suspected and confirmed cases and the setup of health isolation sections.
(c) Suspension of meetings with relatives.
(d) Suspension of treatment activities such as external work, sport, leisure activities and school.
(e) Medical information and adoption of PPE and cleaning procedures.
(f) Close cooperation with the local health department to monitor the outbreak evolution in prison.
(g) Open access to phone and Skype communication.
(h) Employment of prisoners in surgical mask manufacturing.
(i) Limited prisoner displacements.

**Table 1:** Summary list of actions taken by the Italian Prison Service.

Social Distancing	Physical Barriers/ Sanitizing	Movement’s Reduction	Information and Psychological Support
Packages preferably from express courier (not from relatives)	Clean and sanitize frequently	No admission to visitors and relatives	Upload data to DAP regarding infected prisoners and staff
Medical examination and swab in isolated places	PPE also for authorized visitors	Successive admission but with restrictions and controls	Inform local health authorities if released symptomatic
Different pathways during meeting; Different cells for precautionary isolation	Sanitizer dispensers	Stop to movements to other facilities, to Court etc.	In prison classes for instructions on PPE and COVID-19
Different entry/exit	PPE for prison staff (FPP2, gloves etc.)	Reduce or stop treatment activities if infection rates exceed 2% or 5% rates	Facial mask production
Visits to prisoners (when allowed) must be booked	Plastic barriers for meetings		
Different rooms or spaces for meetings			
At least 2 m distance between different meeting groups			
Install tents for pre-triage			
Increase phone and Skype contacts			
Establish sections for healthcare and precautionary isolation measures			

The rate of infected people in Italian prisons, after the first outbreak peak (February–July 2020), was determined to comprise 0.5% of the population in July 2020 by the Antigone’s XVI Report on Prison Conditions [20] (on 7 July 2020, data show a total amount of 287 infected prisoners over a 53,619 population—methodology not shown), and it is remarkably like the national rate for the same period that has been confirmed at 0.4% (infection rate of cumulative cases in Italian population) [22]. 

By the mid of September 2020, the SPACE 1 report showed that the total amount of inmates and staff who had been infected in Italy was 473 (184 inmates and 289 prison staff) but this figure is the cumulative result of three stock data referring to three precise dates (15 April, 15 June, and 15 September) and is not flow data. 

### 3.2. The International Standards and Recommendations

In March 2020, during the first wave of the COVID-19 outbreak, the World Health Organization published an international guidance entitled “Preparedness, prevention and control of COVID-19 in prison and other places of detention”. The document, addressed to “health-care and custodial staff working in prisons and other places of detention to coordinate public health action in such settings”, declares three main objectives. The first one is to guide state parties to draft or to fit their health contingency plans to face the COVID-19 threat in prison. The second objective is to illustrate valid actions to mitigate the risk of spreading the disease in prison and the third one is to help fit the national/local emergency plans to prison health system. The document provides a list of recommendations that comprise the framework within which every decision or choice of any contingency plan must be held. 

We therefore performed a comparison between the WHO recommendations for “planning principles and human rights…” and the Italian prison service guidelines and prison regulations (see Table 2) that allowed us to point out they were mostly satisfied already, with the only significant exception concerning the following:

“Refined allocation procedures should be considered that would allow prisoners at highest risk to be separated from others in the most effective and least disruptive manner possible and that would permit limited single accommodation to remain available to the most vulnerable”.

This issue has not been addressed in Italian guidelines because, most likely, of the awareness that Italian prisons overcrowding would have not allowed much room for manoeuvre. In fact, in the first days of January 2020 the number of inmates was 60,769 (on 1 March it was 61,230 [23] and the capacity of prison facilities was 50,688, with an overcrowding of 119.9% on 1 January 2020 [6].

Prison overcrowding has been partially reduced in the following months by legislative provisions that allowed the freeing of short-term prisoners: on 15 April 2020, prison density was 108,6%, while by 15 June 2020 it was 106% with a slight increase on 15 September 2020, when it was 107.1% [6].

**Table 2:** Comparison between expected/observed interventions (Compliant—C; Non-Compliant—NC; Data not available—NA).

WHO Recommendations on COVID-19 in Prison Management	COMPLIANCE CHECK of Italian Legislation Rules and DAP-Guidelines	
“The provision of health care for people in prisons … is a State responsibility”	Standard met by existing laws	C
“People in prisons and other places of detention should enjoy the same standards of health care…”	Standard met by existing laws	C
“Adequate measures should be in place to ensure a gender-responsive approach in addressing the COVID-19 emergency…”	Standard met by existing laws	C
“Prisons and other detention authorities need to ensure that the human rights of those in their…”	Standard met by existing laws	C
“Enhanced consideration should be given to resorting to non-custodial…”	Standard met by emergency laws	C
“Similarly, refined allocation procedures should be considered that would allow prisoners at highest	Not compliant	NC
“Upon admission to prisons and other places of detention, all individuals should be screened…”	Requirement met by DAP-guidelines	C
“The psychological and behavioural reactions of prisoners…”	Requirement met by DAP-guidelines	C
“Adequate measures should be in place to prevent stigmatization…”	Requirement met by DAP-guidelines	C
“People subjected to isolation for reasons of public health protection, in the context of prisons…”	No evidence found	NA
“Any decision to place people in prisons and other places of detention in conditions of medical isolation should always be based on medical…”	Requirement met by DAP-guidelines	C
“People subjected to isolation for reasons of public health protection, in the context of prisons and other places of detention, should be informed…”	Requirement met by DAP-guidelines	C
“Adequate measures should be in place to protect persons in isolation from any form of ill treatment…”	Standard met by Law—Requirement met by DAP-guidelines	C
“The COVID-19 outbreak must not be used as a justification for undermining adherence to all fundamental safeguards…”	Standard met by Law—Requirement met by DAP-guidelines	C
“The COVID-19 outbreak must not be used as a justification for objecting to external inspection of prisons…”	Standard met by Law—Requirement met by DAP-guidelines	C
“Even in the circumstances of the COVID-19 outbreak, bodies of inspection in the above sense should have access to all people…”	Standard met by Law—Requirement met by DAP-guidelines	C
Appropriate contingency plans, including checklists, should be established to help prison and detention systems to self-assess and improve their preparedness for responding to COVID-19.	Not compliant	NC

The other WHO recommendations that could not be satisfied regard the following issue: “To manage a COVID-19 outbreak, there need to be effective planning and robust collaborative arrangements between the sectors…” and also “appropriate contingency plans, including checklists, should be established to help prison and detention systems to self-assess and improve their preparedness for responding to COVID-19…”. In fact, there is no evidence of such contingency plans for biological events in Italian prisons [24] and that is why, in the second part of this work, we proposed a draft “Contingency plan guideline for bio-event”.

### 3.3. A Proposal for “A Guideline for Prison Contingency Plan in Case of Bio-Events”

According to the experience of other Countries as England-UK (see Multi-agency contingency plan for the management of outbreaks of communicable diseases or other health protection incidents in prisons and other places of detention in England—second edition 2017 [21]) such a plan should be approved and issued by the higher authorities of the prison department, on one hand, and public health, on the other. The plan could also be shared with the departments responsible for civil protection and public security, in addition to any other participating bodies that could be involved in case of need. In Italy the main actors could be the Department of Prison Administration and the General Directorate for health prevention of the Ministry of Health. In case of a malicious, biological attack (e.g., terrorism, organised crime, etc.) Civil Defence could assume a leading role in managing the event. Therefore, it should be appropriate to share the plans with the local prefect.

The plan is intended to:

(a) Guide people, during the first frantic moments, to identify roles and responsibilities and to manage the crisis with a proper, “certified” and approved outline.
(b) Establish how to detect and notify those relevant any incident or outbreak immediately or as soon as possible. Possible tools to improve this type of passive surveillance could be represented by new first responders oriented user-friendly apps for epidemiological modelling [25].
(c) Evaluate the threshold above which the plan must be activated.
(d) Proceed with a risk assessment at the beginning as well as the developmental phases of the incident/outbreak.
(e) Put in place all the shared and agreed solutions to mitigate the risk (prevention and control).
(f) Arrange the logistic and technical network to facilitate internal and vertical communication to “the centre” and the horizontal communication to the prison and to the external community.
(g) Address the communication of the incident/outbreak to the surveillance network represented by, first, the local ASL. and hence to the CCM (National Centre for Disease Prevention and Control) that is the connecting and coordinating service between the Ministry of Health and the local departments in case of surveillance, prevention and prompt response during emergencies.

The plan should be activated after a threshold has been exceeded as follows:

(a) More than two prisoners or prison staff refer and show the same symptoms of an infectious disease and they have shared the same physical spaces at the same time.
(b) The communicable disease is spreading in a faster way or at a higher rate among the prison community with respect to the outside population.
(c) A single prisoner is experiencing the symptoms of a rare and harmful disease such as diphtheria, polio, haemorrhagic fever (like Ebola), botulism, anthrax, cholera, brucellosis, acute Hepatitis A, B or C, salmonellosis, SARS and MERS, tetanus, typhus etc.

The prison governor must be informed immediately of any suspected cases by the medical or custodial staff as well as by self-declaration. They have the responsibility to evaluate the above-mentioned indicators and to activate the plan if necessary (e.g., the biological agent is able to spread among the prison quickly; it could severely affect prison safety and security; there are more than one single infectious hot spots; the possible outbreak could easily spread and harm the outside population; the bioagent is rare and very harmful; there could be a malicious intention behind the disease—bioterrorism etc.). Along with the medical notification to the ASL. (local health department), that is mandatory for health care workers, the prison governor could contact the local health authorities for a timely analysis of the consequences of a possible outbreak.

The declaration of outbreak and the activation of the plan must be communicated to the Regional Prison Administration (Provveditorato), immediately.

#### 3.3.1. Key Roles in the Contingency Plan

The main and necessary professional figures should be the Prison Director, the Prison and Probation Police Commander, the prison health service responsible and the Occupational Doctor. They could form the Incident Prevention Control Team (IPCT) chaired by the director or his/her delegate. For every member of the team a representative should be designated. The team could also be extended to other experts like the prison accountant, the responsible of prison educators, a specialist doctor (e.g., virologists, epidemiologists etc. from the A.S.L), and, eventually, specialized professionals from the prison administration department like architects, civil engineers, and biologists and I.T. experts from the prison and probation police technicians. If necessary, the team should be integrated with other members coming from the Ministry of the Interior (the local Prefect or his delegate) and a representative of Civil Protection if the outbreak/incident is going to exceed the guard levels and endanger the population. Finally, it is valuable to indicate a responsible member of Secretariat and Logistic services (a dedicated room, telephones, computers, vehicles and secretarial staff to type and store reports, letters etc.). In fact, every IPCT meeting must have minutes taken and the chairperson has the responsibility of planning and calling the meetings and to deliver the approved duties to the participants within twelve hours after each meeting. Among the IPCT it is necessary to nominate a responsible for all press communications. If it is not possible to find a suitable professional among the IPCT it would be preferable to recruit a media expert in the Department of Prison Administration (DAP). The communication expert will be the only one authorised to release interviews and press releases except if the IPCT decides otherwise. The interventions will be informative, brief, transparent, respectful of privacy and of legality, empathic and timely. Media, social media included, must be considered.

#### 3.3.2. IPCT Operability

The aim of the IPCT is to deliver operative solutions to the prison director to:

(a) Investigate the presence, nature, extent, rate of infection of the outbreak/biological incident.
(b) Evaluate the scientifically accepted means to stop or reduce the spread through behaviours, engineering (e.g., plastic barriers; routes, fabric buildings, cleaning, water purification, etc.), chemicals (e.g., Disinfectants, decontaminants, soaps etc.), drugs, vaccines, PPE etc.
(c) Assess and classify possible, suspected, and confirmed cases.
(d) Establish the on-site care vs the hospitalization according to (a) outbreak/incident type and entity, (b) clinical evaluation of the disease, (c) possibility to dedicate and set up a section as isolation area (d) availability of ambulances and isolation stretchers (like N-36 isolator) in case of need, or other suitable vehicles.
(e) Decide, record and assign, to the members of the IPCT, responsibilities and means for the realization of specific tasks.
(f) Provide an updated risk assessment that shows how mitigating activities are working.
(g) Find out how to rapidly detect and contain new infectious hotspots in the facility.
(h) Investigate the bio-cluster expansion through contact tracing (included those prisoners who have been released).
(i) Decide the information to give to press agencies/media.
(j) Inform the higher authorities of the DAP—Ministry of Justice, the local and National Health Service (ASL—Regional Department and Ministry of Health) and other stakeholders.
(k) Check the availability of intensive care units near the prison facility.
(l) Establish data sources that can be shared among the IPCT actors with no legal prejudice.
(m) Verify and state the end of the emergency.
(n) Draw up a final report within two months after the end of the emergency.
(o) In case of malicious attack and bioterrorism the prison and probation police.

The commander is responsible for the preliminarily investigation in accordance with the prosecutor. In this case, the IPCT would be integrated by the prefect and by a by representative of the firefighters department. Moreover, the incident scene, that could also be a crime-scene, should be secured by trained and specialised prison and probation police officers and firefighters incident commander should manage the injured rescue, the detection of the agent, the sample collection and the cleaning up of the area.

#### 3.3.3. Audits of the Plan

The first version of the plan shall be issued in its version Rev. 0. Successive issues shall be indicated as Rev. 1, Rev. 2, …, Rev. *n* and changes shall be listed at the beginning of the document. At least every two years, a “first party” audit shall be carried out on the document to verify it is applicable to the current structure of the prison facility and to the local health authorities and moreover to assess its compliance to the up-to-date safety and security standards laws.

The Audit Team should be comprised of two health and security experts coming from the administrations of Health and Justice. They must be persons other than the plan’s authors.

#### 3.3.4. Checklists

It would be straightforward to use defined checklists (Table 3, Table 4, Table 5, Table 6 and Table 7) to manage all the stages of a biological incident emergency and in particular to record IPCT meetings and reports on the one side, to record the timeline of each single step during the emergency time, to record every single IPCT action and to record epidemiological data for inmates and staff. The following tables exemplify a draft model of such checklists:

## 4. Discussion

The COVID-19 emergency has imposed significant Italian government regulations to deal with the harmful outbreak. The fight against COVID-19 in prison in Italy, as in other European countries, is composed of general preventive measures to avoid or at least mitigate the pace of infection and healthcare measures to take care of infected inmates and staff. An overall decrease of the prison population rate has been documented in most state parties that belong to the Council of Europe during the first period of the SARS-CoV-2 outbreak that roughly spans from January to September 2020. The decrease was generally due to prisoners’ release as a preventive strategy to mitigate the infection risk in prison. Secondary, it can be hypothesized that general confinement of populations due to national lockdowns, especially in the first months of the pandemic (from March to June 2020) has had a synergic effect in decreasing the number of crimes [5]. More specific measures were put in place by each prison administration to guarantee healthcare in prison and the respect of rules and of the human rights at the same time [18]. Our study shows that the Italian prison system had already deployed the majority of counteracting measures that WHO proposed as the gold standard for every Country, and this might have contributed to contain the spread of COVID-19 among the prison population.

For example, the decision to reduce the prison density and the great effort that has been put in place by the Italian prison administration satisfying the majority of WHO recommendations have probably proved successful as at the end of the first phase of the outbreak the COVID-19, infection rate in Italian prisons was reasonably comparable to the outside population. Nonetheless, that is a rough inference because of the absence of solid flow data about prison infections during that period and, moreover, the absolute number of infections in 28 European administrations does not seem to correlate with the appliance of the only preventive measure of prisoners’ release [5].

Furthermore, the outbreak emergency has highlighted the need to establish a more robust and standardized approach to manage with CBRNe events in prison facilities as recommended by the WHO [18]. This result can be achieved through drafting contingency plans in accordance with general Guidelines that could be provided by the Ministry of Justice. The need of a guideline for contingency plans would help in standardization of local facilities’ plans which would result in document comprehensiveness and robustness by means of a “senior administration” format.

## 5. Conclusions

One specific concern that has peculiar health, safety, security, and legal implications is that of biological outbreaks in confined environments like prison facilities [26], critical infrastructure and strategic sites for public security, as the COVID-19 pandemic showed. Like residential care homes or cruise ships, prisons are enclosed environments with narrow spaces where close contact between people seems unavoidable [27], and where communicable diseases could lead to extremely severe consequences [28,29,30]. Despite these similarities, during the first wave of the pandemic (winter-summer 2020) infectious rates in Italian prisons were lower than in nursing homes where mortality was documented at around 3% (“test confirmed” data was 0.7%), with large differences among Italian regions [31]. This evidence is probably due to, at least, two factors, one possibly related to the different mean age and the health conditions of the two sub-populations; the other likely to be a consequence of the counteracting measures that the Italian prison service has fielded before WHO specific guidelines were defined and adopted. The Italian DAP has been able to manage the outbreak quite successfully limiting the infection rate in prison under values that are comparable to the national rate during the first wave of the pandemic, and anticipating most of the risk mitigation strategies and prisoners’ rights with respect to measures recommended by the WHO. The only relevant exception was the request of refined allocation procedures to separate inmates in different accommodations in accordance with their vulnerability. The persistent and chronic overcrowding of the Italian prisons [5,6], that was estimated to be around 120% of capacity in January 2020 is probably one of the major vulnerabilities of the entire system; it only it creates objective limits for space management (e.g., they prompted consequent requests to the surveillance courts to decide on the suitability of prison for health-vulnerable prisoners during the outbreak) but it also causes the so called “tunnel effect”, derived from the “theory of scarcity”, that limits the confidence that a complex situation can be properly addressed when scarce resources represent a cause of concern (e.g., scarcity of funds, staff, time, space etc.) [31]. Based on the lessons learned from the COVID-19 pandemic and the evidence reported in this paper, we proposed a new model for the Italian prison service that updates the latest available contingency plans for Italian prison services that date back to 2011 [24] and that lack specific provisions for biological threats. The model provides a general scheme to identify roles, responsibilities, operability, and auditing to assure traceability, reliability, and constant improvement to preserve the health of both the prison population and prison and probation police workers from biological threats.

## Figures and Tables

**Table 3:** A checklist to record IPCT meetings and reports.

IPCT Meetings and Reports					
Participants Name, Phone N., Email					
				
				
Date					
Role/Note					
Minute Nr.					
Minute Date					
Signature					

**Table 4:** A checklist to record the timeline of each single step during the emergency.

Timeline of Events					
	Yes/No_Date	Define/Note	Name/Role	Date	Signature
Identified Outbreak					
Biological Attack					
Other Kind Of Biological Incident					
Notification To The Director					
Notification To The Commander					
Notification To Health Authorities					
Notification To the Service Administration					
Prosecutor Informed					
Notification To the Prefect					
Declaration of an Emergency					
IPCT Call					
IPCT Minutes Acquired and Registered					
Declaration of the End Of The Emergency					
Final Report					
Final Report Submission					

**Table 5:** A checklist to record every single IPCT action.

IPCT Actions						
	Yes/No Date	Description (Report Ref.)	Yes/No Date	Description (Report Ref.)	Yes/No Date	Description (Report Ref.)
Bio Agent Detection						
Roles and Tasks Assigned						
Risk Assessment						
Test Confirmation						
Biocontainment						
Communication Strategy						
Judiciary Sample Collection						
Sick Prisoners						
Hospitalization						
Health Isolation						
Movements Limitation						
PPE Information						
Prisoners Information						
PPE Identification						
PPE Distribution						
Contacts Limitation						
Source Identified						
Clusters Identified						
Suspected Infectious Identified						
Suspected Case Defined						
Confirmed Case Defined						
Vaccination						
Drug Delivery						
Cleaning Strategies						
Disinfection Strategies						
Interpersonal Distancing						
Action X						
Action Y						

**Table 6:** A checklist to record the epidemiological data for inmates.

Injured_Infected_Dead Prisoners									
	Day 1	Day 2	Day 3	Day 4	Day 5	Day 6	Day 7	Day 8	Day X
Name/Number									
Start Date/Hour									
Symptoms									
Isolation									
Hospitalization									
Medical Condition									
Contacts Traced									
Date Of Recovery									
Relat. Informed									
Comment									

**Table 7:** A checklist to record the epidemiological data for staff.

Injured_Infected_Dead Staff									
	Day 1	Day 2	Day 3	Day 4	Day 5	Day 6	Day 7	Day 8	Day X
Rank/Name/Number									
Start Date/Hour									
Symptoms									
Isolation									
Hospitalization									
Medical Condition									
Contacts Traced									
Date Of Recovery									
Relatives Informed									
Comment									

## Data Availability

Not applicable.
